# What mental health professionals and organisations should do to address climate change

**DOI:** 10.1192/bjb.2021.17

**Published:** 2021-08

**Authors:** Adam Monsell, Jacob Krzanowski, Lisa Page, Sharon Cuthbert, Guy Harvey

**Affiliations:** 1 Camden and Islington Mental Health and Social Care Trust, UK; 2 South London and Maudsley Mental Health NHS Trust, UK; 3 Sussex Partnership NHS Foundation Trust, UK; 4 Sussex Partnership NHS Foundation Trust, UK; 5 Cumbria Northumberland Tyne and Wear NHS Foundation Trust, UK

**Keywords:** Sustainability, climate change, mental health, psychiatry, carbon footprint

## Abstract

**Aims and method:**

The climate change emergency is also a mental healthcare emergency. We seek to provide a framework for what mental health professionals and organisations should do to make their practice more sustainable.

**Results:**

There are ethical, legal and organisational imperatives to make mental healthcare more sustainable. Mental healthcare must be refocused with an emphasis on prevention, building social capital and community resilience. Patients must be empowered to manage their own mental health. Efficiencies should be found within the system. Low-carbon ways to deliver care must be found, measured and improved upon. Greater adaptability needs to be built into the system to mitigate the impact of climate change. Sustainability should be integrated into training programmes, and good examples of practice shared and celebrated.

**Clinical implications:**

Mental health organisations and individuals must act now to prevent and adapt for the climate and ecological emergency. Sustainable practice is also good practice.

Climate change will increase and alter the burden of poor mental health worldwide. Direct effects, such as floods and droughts, have been shown to worsen a variety of mental health disorders. Indirect effects, such as displacement of populations, are likely to compound this trend.^1^ There are ethical, legal and organisational imperatives to make mental healthcare more sustainable. The climate emergency is also a health emergency, and clinicians have a duty to act as individuals and as part of organisations.^2^ Sustainability is written into the constitution of the National Health Service (NHS),^3^ and the Climate Change Act of 2008 legislated for a reduction in NHS carbon emissions of 80% over 30 years.^4^ More recently, the NHS has made the formal commitment to become carbon neutral by 2040.^5^

Mental health services are estimated to emit 1.47 million tonnes of carbon dioxide annually.^6^ We must act now to reshape services, both to meet the ambitious targets set by the NHS, and to avoid runaway consequences.

The COVID-19 pandemic has required us to think differently about how we deliver mental healthcare, and provides an opportunity to enact changes now consistent with sustainable future.^7^

## What should people and organisations do?

The Consensus Statement on Sustainability in Mental Health establishes four tenets of practicing mental healthcare sustainably (Box 1).^8^ These focus on reducing greenhouse gas emissions and enabling organisational, social and cultural factors that will help patients achieve better mental health in the future. A health service that targets its resources and empowers patients to take ownership of their health will become more efficient, resilient and less carbon-intensive.

Box 1 Tenets of sustainable mental healthcare.

1. Prevent mental illness, build social capital and promote individual, social and community reliance and mental well-being.
2. Empower patients, staff and carers to manage their mental health.
3. Eliminate wasteful activity.
4. Make use of low-carbon alternatives.

### Focus on prevention, build social capital

Prevention protects resources, and hence saves carbon. Preventative healthcare is more cost-effective than a reactive approach. This is true in mental healthcare, where preventative programmes have shown to be effective in a wide range of common mental health problems such as depression, anxiety and first-episode psychosis.^9^ Prevention of conduct disorders could save £150 000 per case in lifetime costs.^10^ Early intervention programmes can delay or prevent transition to psychosis among participants,^11^ and have significant cost-savings.^12^ Some preventative measures are outside the purview of psychiatric services. Adverse childhood experiences increase the risk of depression by four times, and suicide attempts by 30 times,^13^ yet mental health services are not involved at the advent of these life events. Greater joint working between public service bodies would achieve better long-term mental health outcomes.

Addressing the social determinants of poor mental health can also achieve better outcomes. The Marmot Review established that ‘the lower one's social and economic status, the poorer one's health is likely to be’.^14^ Organisations should make targeted us of resources in areas such as housing, social isolation and employment, to work to improve social capital.

#### Housing

Homelessness and long-term mental illness are linked.^15^ Models such as Housing First have been shown to help individuals engage better with treatment programmes, doing this at around half the cost of traditional models.^16^ The Critical Time Intervention programme can also be effective in preventing homelessness on discharge from in-patient care.^17^

#### Isolation

People with mental ill-health are more likely to suffer adverse consequences of social isolation.^18^ Recent lockdown conditions have shown the impact of social isolation on previously well-managed mental health conditions.^19^ Befriending services can be effective in improving depression,^20^ and is highly valued by patients. Social prescribing can also help address isolation, loneliness and inactivity.

#### Employment

Unemployment is associated with poor mental health,^21^ whereas being in employment or volunteering promotes better mental health.^22,23^ Individual Placement Support services have demonstrated successes, when used, by integrating employment specialists into community teams to support those with severe mental health problems into work.

Addressing social determinants of mental health will enable populations to become more resilient to the effects of climate change. Groups with less social capital are both more likely to experience poor mental health,^14^ and more vulnerable to the effects of climate change.^24^ Conversely, a better housed, stably employed, socially connected population will require less mental healthcare as circumstances change.

### Empower patients to manage their own mental health

A variety of opportunities exist to enable patients to take a leading role in the management of and recovery from their conditions (Box 2).

Box 2 Sustainability within a management plan.

Dr Alvarez, has been reviewing John, a 57-year-old man with depression, who drinks alcohol regularly. John has been calling the ambulance service and police when intoxicated, resulting in frequent visits to his flat. Emergency services suspect that he is doing this in part because he is lonely and isolated. John has said he ‘doesn't do much’ in the day, and his care coordinator confirms he goes to the shop twice a week but nothing else. Dr Alvarez asks what John has enjoyed before – he says he remembers helping his father in the garden but ‘of course, I don't have a garden now’. John discussed him at the team meeting and hears from the occupational therapist about ‘Men in Sheds’, a local gardening group coordinated by peer-support workers. Dr Alvarez contacts John's general practitioner to establish contact with a link worker from the local social prescribing initiative, who meets with John to enable him to attend. After 4 weeks of attending together, John feels ready to go himself and says it is the highlight of his week. Dr Alvarez asks John to monitor his symptoms via an online symptom tracker, and is able to demonstrate to John that he his mood has improved, and alcohol consumption reduced. He is no longer calling emergency services. Dr Alvarez and John discuss a keeping well plan, getting John to identify what has got him better. They agree to meet again in 3 months via video conferencing to review his progress.

#### Co-production

If done correctly, individual, jointly developed care planning forms an opportunity identify what works for patients correctly first time, and hence reduce wasted effort. This needs to be done in a non-tokenistic way that does not marginalise patients.^25^ Co-production must also form an integral part of any service redevelopment.^26^

#### Self-monitoring

Technology can empower patients to manage their own symptoms. Tracking their symptoms online or via smartphones has been shown to benefit a variety of disorders, including anxiety, stress, alcohol and sleep disorders, depression, suicidal behaviours and post-traumatic stress disorder.^27^ Use of symptom monitoring is well developed in Improving Access to Psychological Therapies, and could be expanded to other services.

#### Peer support

Peer support provides excellent opportunities for patients to take ownership of their mental health. They can reduce in-patient admissions across a variety of diagnoses,^28^ and can also link patients to a social support network.^29^

#### Social prescribing

Social prescribing is a key component of personalised care,^30^ and provides for an opportunity to tie together many aspects of sustainable healthcare practice. It has potential to lower the carbon footprint of healthcare by empowering people to pursue their own non-pharmacological solutions to their social, practical and emotional problems.

#### Green and blue space

Access to green and blue space can promote mental health and improve symptoms in a variety of mental and physical disorders.^31^ Green space and horticultural therapy projects should be part of any hospital build or redesign, and Trusts should identify areas where they could form part of their existing portfolios. Green walking groups^32^ and activities centred on blue space both have benefits.^33^ Importantly, those with poor mental or physical health are least likely to have access to green and blue space,^34^ and efforts will be needed to engage these groups.

### Eliminate wasteful activity

Wasteful activity is a significant contributor to both the carbon footprint and financial cost of the NHS. A leaner, smarter service will deliver higher-value, more sustainable healthcare.

#### Medicine optimisation

Although the carbon impact of psychiatric prescription is currently poorly understood, some quick wins exist. Using long-acting injectable medications at the longest evidenced-based interval rarely occurs, but could reduce the cost of prescribing by £250 per patient per year, or a total of 170 000 kg carbon dioxide equivalent.^35^ Psychiatry also has considerable influence over prescribing in primary care, and should routinely work with general practitioners to reduce pharmaceutical waste, polypharmacy and overprescribing. There should be greater use of structured medication reviews to work with patients to optimise their medications.^36^ These form further opportunities for shared decision-making over treatment.

#### Concordance and treatment effectiveness

Half of all medicines dispensed are not taken as directed.^37^ In mental healthcare, the figure is likely to be even higher. Better understanding the reasons for non-adherence, such as side-effects, personal beliefs or other barriers, may go some way to reducing waste. There may also be opportunities for patients to pursue treatment strategies that de-emphasise the role of medication, if circumstances permit. The open dialogue approach has been successful both in treating symptoms of psychosis and returning patients to work, largely in the absence of medication;^38^ its efficacy in the UK is currently being evaluated. In some situations, it may be possible to continue to support people with severe mental illness who choose to not take medications at all; so called ‘managed non-adherence’.

#### Reducing ‘did not attend’ rates

‘Did not attend’ rates in mental healthcare are estimated to be between 15 and 20% higher than other specialties.^39^ Patients who miss appointments tend to be more unwell, and have a higher chance of relapse and hospital admission.^40^ A reduction in missed appointments can be obtained by gaining a better understanding of their cause, and adapting to this in ways relevant to patients.^41^

### Make use of low-carbon alternatives

**Fig. 1:**
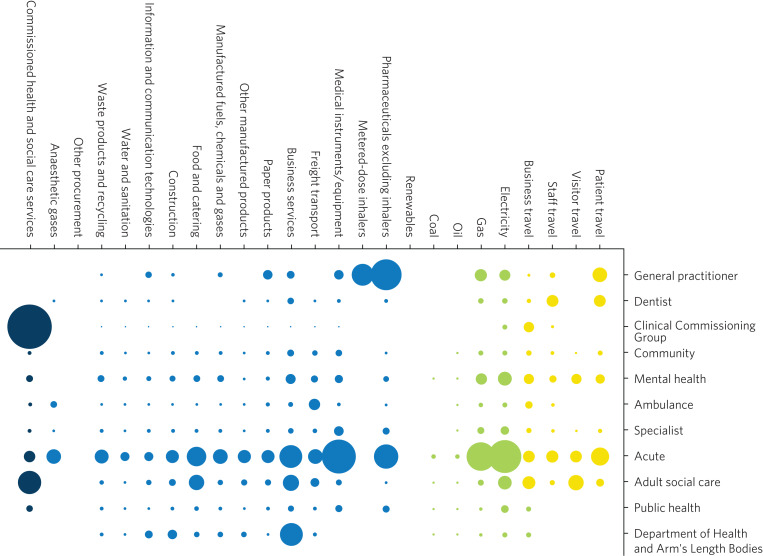
Mental healthcare carbon emissions compared with other healthcare sectors. Relative carbon emissions of healthcare sectors (kgCO2e).

Mental healthcare emissions are more evenly spread across a variety of these direct and indirect sources than in other specialities (Fig. 1).^5^ The lack of a quick fix reinforces the need for collective action by individuals across mental health organisational divisions.

#### Low-carbon treatment choices

Clinicians need to be aware of the carbon impact of their treatment choices, and offer low-carbon treatment choices when possible. There is a need for a better understanding, and the NHS should work with pharmaceutical companies to make this readily available for clinicians. Meanwhile, evidence-based alternatives, such as electronic cognitive–behavioural therapy and other web-based psychology programmes, are available for disorders such as depression,^42^ anxiety^43^ and insomnia.^44^ These have as little as a fifth of the carbon emissions of face-to-face cognitive–behavioural therapy (230 kg *v*. 1100 kg), and a quarter of the carbon emissions of a course of antidepressant treatment with psychiatrist follow-up (900 kg).^45^

#### Active transport and reduction in vehicle emissions

Staff and patient travel forms a significant component of mental healthcare carbon emissions. Clean and zero emissions fleet vehicles, cycling and other types of active travel are all part of the solution; mental health organisations must take steps to procure and promote these. The 2019 Royal College of Psychiatrists sustainability prize winners from Southern Health NHS Foundation Trust ran a project to reduce their transport emissions, using online meetings, cycling or walking, and car sharing, saving 22 216 kg carbon dioxide equivalent over a year.^46^

#### Energy use

Mental health organisations should invest in smart energy systems, and to procure their energy from the greenest possible sources. By installing a piece of software that remotely shut down computers not in use overnight, NHS Oldham was expected to save over 800 000 kg carbon dioxide equivalent and £41 000 in the first year.^47^ Clinicians can identify energy-saving schemes in their workplaces. The Centre for Sustainable Healthcare run the Green Ward competition, offering guidance and support for sustainable schemes, including energy use.

#### Waste and recycling

Although a relatively small component of the overall carbon impact of the NHS, interventions involving waste often are the most definable and easily engaged-with green programmes. The Royal Surrey Hospital's recycling programme grew from a group of nurses carrying home recyclable waste into a dedicated recycling centre for the Trust, and 60 ‘sustainability champions’.^48^

#### Food and catering

Mental health organisations must work to offer their in-patients and staff members sustainable and healthy food options. North Bristol NHS Trust successfully worked with their wholesalers to source all of their ingredients within a 50-mile radius.^47^ Clinical staff can do much via feedback to on-site catering options to include more sustainable options, or taking steps to change their own diet at work.

### Plan, measure and improve

Transitioning toward sustainable models of care requires unprecedented coordination between and within mental health organisations and their local partners. Success depends on a structured, coordinated strategy and ways of measuring and improving changes.

#### Green Plans

All mental health Trusts are required to have a board-approved Green Plan, but their importance to organisations currently varies. Sustainability should be an integral part of mental health organisations’ strategic approach and should have executive-level buy-in. An effective Green Plan will be led by a Board member and have wide representation. The Sustainable Development Unit and NHS England have published guidance on how to develop a Green Plan.^49^

#### Carbon footprint

The Sustainable Development Unit has tools for NHS Trusts to measure and reduce their carbon footprint in procurement, and have forthcoming plans for other divisions. An ‘ethical procurement’ tool is also available. The Royal College of General Practitioners has produced a ‘Green Impact for Health Toolkit’, enabling general practices to audit their practice – a similar toolkit should exist for mental healthcare organisations.

#### Quality improvement

**Fig. 2:**
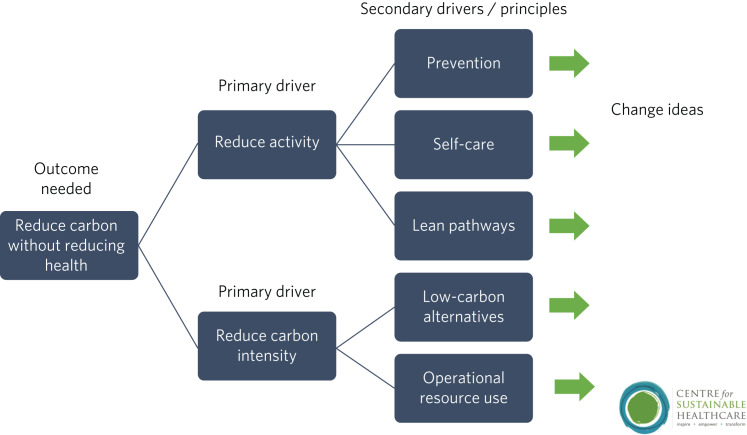
Applying sustainable clinical practice principles in quality improvement. Published from the Centre for Sustainable Healthcare under creative commons licence CC BY-SA 4.0.

Quality improvement is an effective framework to achieve sustainability aims. One advantage of the quality improvement model is that it seeks to understand local systems first, making it more effective at solving local problems. It is also a ‘bottom-up’ approach, fostering a sense of ownership and ambition among staff. The Centre for Sustainable Healthcare have adapted the quality improvement model for sustainability, which can be used in mental healthcare settings (Fig. 2).

### Adapt the system

Climate change will bring about longer-term shifts in patterns of need, mediated through changing populations, economic hardship, increased social division^1^ and poorer physical health.^50^ In the UK, climate change is leading to more flooding and heatwave events, which have adverse impacts on mental health.^51–53^ In the global South and elsewhere, additional hazards such as wildfires, droughts, hurricanes and cyclones, are recognised drivers of poor mental health,^54^ particularly for those with pre-existing difficulties.^55^

Clinicians must prepare for this change and increase in mental disorder. There will need to be flexibility built into the system, as the full effects of climate change on the population are unknown.

### Equipping psychiatrists to become sustainable practitioners, now and in the future

Organisations must play the central role in directing a shift in the organisation and delivery of clinical services. However, the normalisation of such practice through education, knowledge sharing via networks, and empowerment, is essential to allowing clinicians to play their part.

#### Training

There is broad interest among psychiatry trainees and medical students in becoming sustainable practitioners, but many do not see it as a core feature of their role as trainee doctors. Sustainability should be integrated into training and established as a central responsibility of a psychiatrist. Medical schools such as Lancaster Medical School are already embedding sustainability into their curriculum,^56^ and sustainable practice should be made part of e-portfolio and annual review of competency progression commitments. There are also broader arguments to place sustainability at the heart of the General Medical Council's duty of a doctor Gold Guide.

#### Sharing good practice

Attendees at conferences and meetings should routinely expect to see a focus on sustainable healthcare, as has been the case at several regional conferences to date. Video conferencing opens up more opportunities to do this in a sustainable way. Networks such as PsySustnet provide an additional resource to exchange learning. Awards such as the Royal College of Psychiatrists’ annual sustainability prize have successfully celebrated outstanding achievement in sustainable mental healthcare.

#### Empowering employees

Mental health organisations should enable their employees to engage with sustainable activities, and bring their knowledge and energy to their workplaces. The psychiatry higher training programme has integrated ‘special interest’ time, where trainees can pursue projects to the benefit of patients, Trusts and trainees alike. This could be widened to other mental health practitioners to allow all to engage in sustainability projects. A central part of empowerment should also support the growing understanding of supporting the well-being of clinicians.

## Summary

Mental health organisations must act now to prevent and adapt for the climate and ecological emergency. Clinicians’ involvement is vital for developing effective and safe sustainable models of care. There is much work to be done, and there are significant changes to be made to the system to adapt to these needs. The good news is that much can be achieved through prevention of illness, choosing low-carbon treatment strategies, controlling waste and empowering patients to be more in control of their care and treatment. The best news is that sustainable practice is also good practice.

## About the authors

**Dr Adam Monsell** is an ST5 in substance misuse services at Camden and Islington Mental Health and Social Care Trust and sustainability scholar at the Royal College of Psychiatrists, UK. **Dr Jacob Krzanowski** is an ST6 in substance misuse servcies at South London and Maudsley Mental Health NHS Trust and an associate at the Centre for Sustainable Healthcare, UK. **Dr Lisa Page** is a consultant psychiatrist in liaison services at Sussex Partnership NHS Foundation Trust, UK and Clinical Senior Lecturer in Medical Education & Psychiatry at Brighton & Sussex Medical School, UK. **Dr Sharon Cuthbert** is a consultant psychiatrist in adult inpatient services at Sussex Partnership NHS Foundation Trust, UK. **Dr Guy Harvey** is a consultant psychiartist in adult inpatient services at Cumbria Northumberland Tyne and Wear NHS Foundation Trust, UK.
